# A Digital-First Health Care Approach to Managing Pandemics: Scoping Review of Pandemic Self-triage Tools

**DOI:** 10.2196/40983

**Published:** 2023-05-17

**Authors:** Christina Ziebart, Marisa L Kfrerer, Meagan Stanley, Laurel C Austin

**Affiliations:** 1 Department of Physical Therapy Faculty of Health Sciences Western University London, ON Canada; 2 Department of Occupational Therapy Faculty of Health Sciences Western University London, ON Canada; 3 Western Libraries Western University London, ON Canada; 4 Ivey Business School Western University London, ON Canada

**Keywords:** pandemic, digital, scoping review, health care, triage, self-triage, self-assessment, patient navigation, mobile phone

## Abstract

**Background:**

During the COVID-19 pandemic, many patient-facing digital self-triage tools were designed and deployed to alleviate the demand for pandemic virus triage in hospitals and physicians’ offices by providing a way for people to self-assess their health status and get advice on whether to seek care. These tools, provided via websites, apps, or patient portals, allow people to answer questions, for example, about symptoms and contact history, and receive guidance on appropriate care, which might be self-care.

**Objective:**

This scoping review aimed to explore the state of literature on digital self-triage tools that direct or advise care for adults during a pandemic and to explore what has been learned about the intended purpose, use, and quality of guidance; tool usability; impact on providers; and ability to forecast health outcomes or care demand.

**Methods:**

A literature search was conducted in July 2021 using MEDLINE, Embase, Scopus, PsycINFO, CINAHL, and Cochrane databases. A total of 1311 titles and abstracts were screened by 2 researchers using Covidence, and of these, 83 (6.76%) articles were reviewed via full-text screening. In total, 22 articles met the inclusion criteria; they allowed adults to self-assess for pandemic virus, and the adults were directed to care. Using Microsoft Excel, we extracted and charted the following data: authors, publication year and country, country the tool was used in, whether the tool was integrated into a health care system, number of users, research question and purpose, direction of care provided, and key findings.

**Results:**

All but 2 studies reported on tools developed since early 2020 during the COVID-19 pandemic. Studies reported on tools that were developed in 17 countries. The direction of care advice included directing to an emergency room, seeking urgent care, contacting or seeing a physician, being tested, or staying at home and self-isolating. Only 2 studies evaluated tool usability. No study demonstrated that the tools reduce demand on the health care system, although at least one study suggested that data can predict demand for care and that data allow monitoring public health.

**Conclusions:**

Although self-triage tools developed and used around the world have similarities in directing to care (emergency room, physician, and self-care), they differ in important ways. Some collect data to predict health care demand. Some are intended for use when concerned about health status; others are intended to be used repeatedly by users to monitor public health. The quality of triage may vary. The high use of such tools during the COVID-19 pandemic suggests that research is needed to assess and ensure the quality of advice given by self-triage tools and to assess intended or unintended consequences on public health and health care systems.

## Introduction

### Background

Traditionally, health care systems have been structured as in-person, one-on-one visits between a patient and clinician, leading to a congregation of patients in emergency departments and clinic waiting rooms [1]. This is a problem as viral spread is a concern, especially during a pandemic. A potential solution to reduce crowded waiting rooms, reduce the risk of exposure to pandemic viruses, and reduce unnecessary trips to providers for initial triage is to use digital technologies for self-assessment of symptoms and provide advice in nonurgent situations [1-4]. Another notable advantage of using a digital approach to health care during a pandemic is to monitor the epidemiological and clinical characteristics of the virus [5]. At the start of the COVID-19 pandemic, it was unclear which signs and symptoms were associated with the novel virus. Digital tracking is a strategy to better understand the virus and learn how to protect against it [3].

Digital tools have been used in previous pandemics, such as severe acute respiratory syndrome (SARS), Ebola, and influenza and the H1N1 strain of the influenza [6-9]. These studies have focused on surveillance [6], contact tracing [7], case management [7,8], the management of laboratory results [9], and self-triage [4,10]. Over time, with advances in technology and greater accessibility to technology, more patient-facing tools have emerged. These have been used to help with patient triage, at-home monitoring of symptoms, self-assessment of disease, and virtual (not in person) consultations with physicians [4,10,11].

The integration of technology is a large component of the response to managing the COVID-19 pandemic. COVID-19 self-triage and self-assessment tools were quickly introduced by public and private entities in many countries. These tools are intended to allow citizens to screen themselves when making health care–based decisions. The goals of such tools include directing care [3,12], such as calling for an ambulance, going to a physician, encouraging self-care when appropriate [13,14], and alleviating some of the demands of the health care system [14,15]. Other benefits of such tools could be consistent triage across all encounters; an ability to quickly update triage decision logic as more is learned about a novel pandemic virus; and an ability to gather data for surveillance, monitoring, and predicting health care demand [16,17].

Triage is defined as a medical screening of patients to determine their relative treatment priority [18]. Digital triage has been defined as a tool that emulates the decision-making ability of a human expert designed to navigate complex triage problems within a health care system on a massive scale using if-then algorithmic branching logic rules [19]. Digital tools allow patients to triage themselves with predetermined logics that then produce prompts for future actions. Traditionally, medical triage is conducted by a health care professional who intakes information about the patient’s health and then determines their priority of care. During the COVID-19 pandemic, many digital tools were designed to allow people to input their own information, and the tool would then assess the priority of care needs and prompt what they should do next. These prompts include providing a direction for the patient to receive care, for example, to call for an ambulance, self-manage, and self-isolate.

### Objectives

This scoping review aimed to explore the literature on self-triage tools that direct or advised care for adults during a pandemic and consider the key outcomes of the studies. We wanted to know where such tools have been implemented; compare ways they direct to care; and understand whether they have been integrated into a defined health system or systems, how they were administered, and key research questions and findings.

## Methods

### Overview

This scoping review used the framework proposed by Arksey and O’Malley [20] and the enhancements proposed by Colquhoun et al [21] and Levac et al [22]. Scoping reviews provide a broad understanding of the literature on a specified topic. They provide a comprehensive overview of the literature, identifying published literature and concepts supporting the research area. A scoping review was selected for this study to gain a broad understanding of the state of the literature on pandemic digital triage tools that direct patient care.

### Data Sources and Searches

MEDLINE, Embase, Scopus, PsycINFO, CINAHL, and Cochrane databases were searched on July 14 and July 15, 2021. These databases were selected because of the nature of their peer-reviewed journal content relating to the research question. We included conference proceedings, preprints, non–English language articles, and any other formats retrieved in our searches. Multimedia Appendix 1 provides a comprehensive summary of the search strategy.

### Study Selection

To capture the self-assessment tools used during the pandemic, the inclusion and exclusion criteria shown in Textbox 1 were applied.

**Figure 1:**
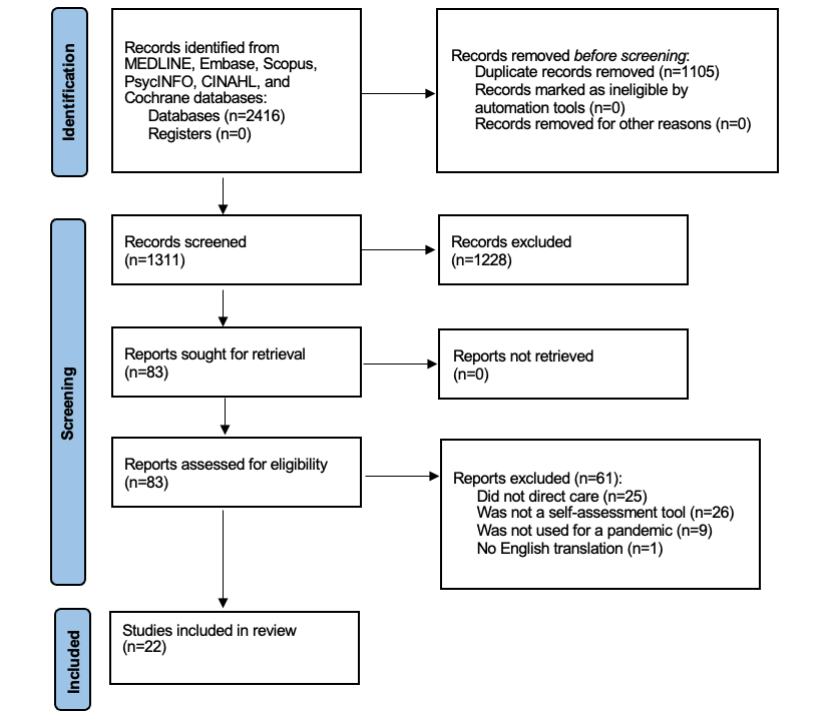
PRISMA (Preferred Reporting Items for Systematic Reviews and Meta-Analyses) flow diagram for study selection.

A total of 22 articles were included in this scoping review (Figure 1 [23] provides a PRISMA [Preferred Reporting Items for Systematic Reviews and Meta-Analyses] diagram detailing the process). After removing 1108 duplicates, 1311 titles and abstracts were screened, resulting in 83 articles for full-text review. Two authors (CZ and MLK) screened all the articles by applying the inclusion and exclusion criteria. If there were disagreements on whether to include an article, the fourth, most senior author (LCA) provided a resolution.

**Inclusion and exclusion criteria for study selection.**

**Inclusion criteria**

- Studies including self-triage tools that direct to care
- Studies conducted during a pandemic
- Studies conducted in adults (aged >18 years)

**Exclusion criteria**

- Studies including tools administered by physician, nurse, hospital staff or administrator
- Studies on telemedicine
- Studies in non-English text where no translation was provided

### Data Extraction

A data extraction form (using Microsoft Excel) was created to extract and chart the following data: authors, year of publication, country of publication, country in which the tool was used, whether the tool was integrated into a health care system, the research question and purpose of the study, the number of users, the direction of care provided, and key findings. Data extraction was conducted by 3 authors (CZ, MLK, and LCA) and verified by 1 of those 3 authors (CZ) by spot checking the data to confirm consistency across the researchers.

### Data Synthesis and Analysis

After charting the data, we assessed the distribution of the articles by publication year and country of origin. Frequencies were used to gain an understanding of the dominant areas of research, in terms of which countries these tools were used, and the countries in which the authors published.

A more in-depth analysis of the literature was conducted to gain a better understanding of where the tools advised them to enter the medical system, whether the tool was used in a defined health care system, and how the tool was administered. A defined health care system was defined as an organization that was in place before the pandemic that provided care or health communications, for example, a hospital, the Ministry of Health, or a public health office. The administration of the tool was defined as how the public gained access to use the tool, for example, via a website or phone app. We also aimed to identify potential gaps in our broad understanding of the use, impacts, and limitations of self-assessment tools during a pandemic.

## Results

### Location of Included Studies

Of the articles included in this review, 12 were published in 2020, 8 were published in 2021, and 2 were published in 2010 to 2011. Tools studied were mostly located in the United States (6/22, 27%) [3,10,11,19,24,25]. A total of 12 studies were conducted in other countries, including France (3/12, 25%) [13,15,26], Iran (1/12, 8%) [27], Denmark (1/12, 8%) [28], China (1/12, 8%) [29], Nigeria (1/12, 8%) [30], Ireland (1/12, 8%) [31], Greece (1/12, 8%) [32], Finland (1/12, 8%) [33], Canada (1/12, 8%) [6], and Switzerland (1/12, 8%) [14]. Furthermore, 4 studies were conducted in >1 country, with 1 study conducted in the United States, India, Nepal, and Bangladesh [34]; 1 study conducted in the United States, Japan, Singapore, and the United Kingdom [12]; and 2 studies conducted worldwide [35,36].

### Description of Tools and Studies

Table 1 provides key characteristics of the tools and Table 2 provides key information on studies reviewed, including study location, whether the tool was integrated into a health care system, research questions or the purpose of the study, sample size or the number of users and uses of the tool, how tools were directed to care, and key research findings.

**Table 1:** Characteristics of the tools (n=22).

Characteristics		Studies, n (%)
**Integrated into an existing health care system**		
	Yes	17 (82)
	No	5 (18)
**Administration of tool**		
	Web-based app	9 (41)
	Website	6 (27)
	Mobile app	4 (18)
	Patient portal	2 (9)
	Stand-alone platform	1 (5)
**Pandemic**		
	H1N1	2 (9)
	COVID-19	20 (91)

**Table 2:** Summary of articles included in review.

Study	Location of tool or tools	Health care system or systems integrated into	Research question or purpose	Direction to care: the tool directs to	Sample size, the number of users, or number of times accessed	Key findings
Azadnajafabad et al [27], 2021	Iran	Yes—Ministry of Health and Medical Education of Iran	To determine the effectiveness of a web-based self-screening platform to offer a population-wide strategy to control the massive influx to medical centers	COVID-19 testing centers, hospital, and medical centers, to be more cautious	310,000 users	Successful implantation and proven potency of such platform suggest more application of telehealth in public health disasters. Details of the platform in this study can be useful for further deployment of similar platforms.
Collado-Borrell et al [35], 2020	Worldwide: 114 applications that were used in several countries	None	To identify and analyze the characteristics of smartphone apps designed to address the COVID-19 pandemic	It varied based on the app.	114 apps	This study found that the greatest number of downloads of self-assessment apps were for those developed by governments, except for the WHO^a^ app. The app with the highest number of downloads was developed by the Indian government, followed by the Polish and Colombian governments. The main purpose of the apps was to provide general information about the pandemic. Mobile apps can be used as a tool for patient communication and monitoring.
Denis et al [15], 2021	France	Yes—French National Health Care System	To assess whether daily reports of anosmia (lack of smell) predicted positive RT-PCR^b^ tests results, daily ED^c^ visits, daily conventional hospitalization, and daily ICU^d^ admissions	ER^e^, primary care, stay home, or use the tool again if symptoms evolve; if severe symptoms, advises to contact a GP^f^ or ED	13,000,343 questionnaires completed from March to November 2020	Peak daily reports of anosmia on the website predicted hospitalizations, ICU admissions, and positive RT-PCR tests. However, in the second wave of the pandemic, this did not hold true. The authors attribute the difference to the fact younger people were affected in the second wave. They conclude numbers with anosmia predict hospital demand for older adults. “Although this tool does not accurately anticipate an increase in the magnitude of hospitalization, it seems to accurately predict the reduction in the hospitalization rate.”
Denis et al [26], 2020	France	Yes—French National Health Care System	To determine if self-reported symptoms could help monitor outbreak dynamics in France	ER, primary care, stay home, or use the tool again if symptoms evolve; if severe symptoms, advises to contact a GP or ED	3,799,535 questionnaires completed	“This study suggests that self-reported symptoms of COVID-19 are correlated with COVID-19–related hospitalizations and that anosmia may be strongly associated with COVID-19.”
Dhakal et al [34], 2020	United States, India, Nepal, Bangladesh	None	They develop an app that takes verbal input to self-assess for COVID-19, then test it with users to study the app’s performance, its usability, and demands on the user’s mental capacity	On the basis of symptoms users are advised to call 911 and visit ER; stay home and contact medical personnel and take over the counter medications as needed	22 users	Users of the novel IVACS^g^ app did not experience high workload to use the tool. Some users experienced frustration as they had to repeat information; the system did not manage all dialects equally well. This did sometimes lead to different results for the same information input.
Galmiche et al [13], 2020	France	Yes—French National Health Care System	To determine if a self-triage tool could reduce the burden on emergency call centers and help predict increasing burden on hospitals	ER, primary care, stay home, or use the tool again if symptoms evolve; if severe symptoms, advises to contact a GP or ED.	3,494,687 questionnaires completed	“The launch of the self-triage web application was followed by a nearly 10-fold increase in COVID-19–related hospitalizations with only a 23% increase in emergency calls, even though the number of completed questionnaires quickly surged, including questionnaires leading to a recommendation to call an emergency call center.” The authors note that they cannot conclude that the application lead to alleviation of demand on emergency call centers.
Hautz et al [14], 2021	Switzerland	Yes—Swiss Federal Office of Public Health	To implement a web-based triage tool targeted at the current pandemic, adapt the content and goals, and assess its effects	Obtain test, call health care provider	17,300 site visitors during the first 40 days	“During the first 40 days of the triage tool’s deployment, the site saw more than 17,300 visitors—69.8% indicated they would have contacted the health care system if the web-based test had not been available”
Heo et al [36], 2020	Developed in South Korea. Translated into 5 languages; available worldwide	No	This study aims to aid the public by developing a web-based app that helps patients decide when to seek medical care during a novel disease outbreak.	10 levels of risk assessed; the highest advised testing; other levels suggested strength of recommendation to test, down to “no evidence of need to test.”	83,640 users in 141 countries during March 2020	An expert-opinion–based algorithm and app for patient screening and guidance can be beneficial in a circumstance where there is insufficient information on a novel disease and medical resources are limited.
Jensen et al [28], 2020	Denmark	Yes—Copenhagen Emergency Medical Services	To track call volumes and track web-based COVID-19 self-assessment tools, and to examine the potential effect of these initiatives on reducing nonessential EMS^h^ call volume and EMS queue time in the ongoing pandemic	Hotline for additional evaluation, self-quarantine and monitor symptoms, educational materials	24,883 users	“The web triage was widely used with more than 107,000 users from its launch. However, no effect on call volume is indicated or documented. Users were mainly younger adults.” “The web triage was limited in interaction, and as expected, not all symptoms were presented; consequently, some potentially infected persons could have been missed.” “The authors find that the web triage might run the risk of being too simple to be useful for some. Furthermore, without revisions, some citizens might not trust the answers owing to the simplicity and rigidity of the first version.”
Jaeger et al [6], 2011	Canada	Yes, integrated into a campus health care clinic website	To develop a tool to ease the burden of H1N1 influenza on a campus clinic by promoting self-care, generating medical notes, and identifying vulnerable students	The resulting screen described steps for self-care along with instructions as to when, where, and how to seek further medical help if needed	1432 users	Integrating the triage tool into a university or campus clinic showed that “real-time influenza surveillance data from a campus community can be achieved by student-initiated, web-based input. This process is invaluable in monitoring influenza activity on campus, providing timely health advice, decreasing unnecessary visits to the campus medical clinic, and assisting the local public health department in valuable surveillance activities.”
Jormanainen and Soininen [33], 2021	Finland	Yes—Finnish government	To describe use, users, and some performance aspects of the Finnish Omaolo COVID-19 web-based symptom self-assessment tool in Finland	Put into 3 major groups: no need for treatment, low or high priority for treatment	1,937,469 questionnaires completed	The Finnish Omaolo COVID-19 self-assessment tool classified users into 3 major groups: no need for treatment, low or high priority for treatment. In total, there were 1,937,469 responses with 220,535 categorized as high priority.
Judson et al [3], 2020	United States	Yes—University of California, San Francisco Health	To rapidly deploy a digital patient-facing self-triage and self-scheduling tool in a large academic health system to address the COVID-19 pandemic	Asymptomatic patients were asked about exposure history and provided relevant information. Symptomatic patients were triaged into 1 of 4 categories: emergent, urgent, nonurgent, or self-care, and then connected with the appropriate level of care via direct scheduling or telephone hotline.	Completed 1129 times by 950 unique patients in the first 16 days	The tool was designed to “have high sensitivity to detect emergency-level illness and high specificity when recommending self-care, both of which were greater than 85%. Despite designing the tool with this conservative approach, the most frequent triage disposition was self-care. Most of these patients did not make further contact with our health system during the subsequent 2 days. This tool may have therefore prevented hundreds of unnecessary encounters.”
Kellermann et al [10], 2010	United States	Yes—Centers for Disease Control and Prevention	To rapidly develop and deploy a digital tool that could help minimally trained health care workers, screen large numbers of patients with influenza-like illness. The purpose evolved to be to create a patient-facing self-triage and self-scheduling tool available on web	ED, contact GP, go to a walk-in clinic, stay home	2758 users retroactively assessed	Tool was implemented by several organizations, including the Centers for Disease Control and Prevention. Authors noted it is possible the tool gave some wrong advice with harm that is unknown. No adverse events owing to use of the tool was reported. Authors reported one estimate that 10,000 unneeded visits to EDs were avoided by users of the tool on one website. Prospective data are needed to understand the tool’s impact further.
Kouroubali et al [32], 2020	Greece	Yes—The Center for eHealth Applications and Services of the Foundation for Research and Technology-Hellas	The purpose of this study was to design a platform, dynamically adapted according to patient preferences and medical history, to support patient-centered information, management and reporting of symptoms related to COVID-19. The platform incorporates modules for citizens, health care providers, and public health authorities to support safety during the current crisis.	Personalized recommendations, communication, position tracing, and public health visualizations	Not reported	The developed platform (ICT^i^), *Safe in COVID-19*, offered a way “for citizens to track their symptoms over time, enhancing a sense of safety during isolation.” The platform showed high user adherence and that users did not need high technology literacy (useful for older adults).
Lai et al [19], 2020	United States	Yes—Mass General Brigham	To use an AI^j^ tool to capture the initial broad screening categories of risk to determine whether the patient required additional consultation with a COVID-19 expert via the Mass General Brigham COVID-19 expert either via the COVID-19 hotline, via an on demand virtual consultation, or in person	Information on what to do if influenza, self-quarantine, asymptomatic, or symptomatic COVID-19. Also provides advice for pregnant women, children, and older people with risk factors.	40,000 questionnaires completed	Implementing a digital prehospital triage system (using AI with a chatbot) helped redirect patient flow and risk factor scoring and eliminating bottlenecks in health care triage. The chatbot was made specifically to Mass General Brigham, which is an academic or integrated health care system. The authors suggest AI as an underused aspect in triage, and through AI, patients will be able to access prompt, evidence-based advice, and direction to the most appropriate care setting.
Lunn et al [31], 2021	Ireland	None	An experimental study to test whether decision aids can support people on when to self-isolate	Self-isolation, call GP, restrict movements for 14 days	500 users	Decision trees or aids in general were shown to support self-isolation during COVID-19. “In all three stages, the interventions generated some statistically significant, positive outcomes. Overall, therefore, the study provides evidence that decision aids can be used to support self-isolation during the COVID-19 pandemic.”
Mansab et al [12], 2021	United States, Japan, Singapore, United Kingdom	Yes—US Centers for Disease Control and Prevention Coronavirus symptom checker; United Kingdom 111 COVID-19 Symptom Checker; Singapore COVID-19 Symptom Checker; Japan Stop COVID-19 Symptom Checker	Using 52 use cases, to compare how likely it is each tool recommends clinical assessment, to ascertain whether they differentiate mild from severe COVID-19 cases, and how well they detect time-sensitive COVID-19 mimickers, such as bacterial pneumonia and sepsis	Stay home, contact a public health preparedness clinic or a GP, go to ED. Stay home or contact medical center. Stay home, call a medical provider within 24 hours, go to ED. Stay home, call telephone triage, call telephone triage, and talk to a nurse, or go to ED.	52 case scenarios were developed and applied to each of the 4 tools.	The tools varied in ability to appropriately advise whether to stay home or go on for clinical advice or assessment, including whether to go to an ED. The United States and United Kingdom tools often advised to stay home when clinical assessment was warranted. All 4 tools failed to advise going to an ED for the case with a form of sepsis.
Morse et al [25], 2020	United States	Yes—Sutter Health System	To evaluate the user demographics and levels of triage acuity provided by a symptom checker chatbot deployed in partnership with a large integrated health system in the United States.	Chatbot directed to 1 of 8 levels of triage advice, which were grouped into 3 levels of acuity	26,646 questionnaires completed	The characteristics and recommendations of the Sutter Health AI symptom checker and chat box offered 8 levels of triage advice. Patient demographics, such as age and health literacy were shown to be important to consider when developing symptom checkers. “Over a 9-month period, we saw robust use, particularly from younger and female users. Just under half of the assessments were completed outside of typical physician office hours, suggesting that there is a significant number of low-acuity concerns for which tailored guidance is not easily accessible during off-hours”
Owoyemi et al [30], 2021	Nigeria	Yes—Nigeria Centre for Disease Control	To build a public-facing tool (Wellvis) and deploy through mobile devices for the surveillance of COVID-19 in Africa and possibly other continents	Direct to 1 of 3 levels: low risk (retake assessment after a few days, safety precautions, health information on COVID-19), medium risk (retake assessment after a few days, observe for indicative symptoms, report to Nigeria Centre for Disease Control) or high risk (self-isolate and immediately report to their respective local disease control agency)	Not reported	Mobile phone apps used for surveillance and reporting on infectious diseases showed the value of citizen participation and offering risk information and possible next steps. This 8-item triage tool showed to be useful for managing COVID-19 and the reporting of symptoms contributed to public health’s ability to understand how to relieve burden on health systems and for prevention and control.
Perlman et al [11], 2020	United States	Yes—K Health Inc	To describe the characteristics of people who use digital health tools to address COVID-19–related concerns; explore their self-reported symptoms and characterize the association of these symptoms with COVID-19; and characterize the recommendations provided by digital health tools	Social distancing, quarantine, isolation, or seeking immediate medical evaluation. Users were also informed if they were at increased risk for COVID-19 complications, and users with risk factors and symptoms were encouraged to consult a physician	71,619 users	After investigating 3 digital health tools on the K Health app to directly manage COVID-19–related concerns, the authors suggested that automated, data-driven digital health tools, as well as remote care provided by a human physician (rather than AI) can help provide health information and guidance during a pandemic. Potential benefits would be to reduce exposure and burden on health care system.
Runkle et al [24], 2021	United States	Yes—Buncombe County Health and Human Services	To assess a participatory surveillance system. The study seeks to examine whether participatory surveillance efforts can aid local health officials in predicting and understanding COVID-19 activity in the community	Call 911, stay home, connect with health care provider, get tested, self-monitor	1755 users	A public health COVID-19 self-checker was shown to be a low-cost and flexible strategy to collect surveillance data on local changes in COVID-19 symptoms and to be used to monitor the efficacy of public health responses. The tool also “provided a means for local health officials to understand how many people with COVID-19 symptoms were in contact with a health care provider, were tested, and frequently encountered barriers to accessing health care and testing resources”
Yu et al [29], 2020	China	None	To assess a smartphone COVID-19 self-triage app	Influenza symptoms: stay home and care for self; self-quarantine if exposed to COVID-19 disease; seek medical treatment if experience COVID-19 symptoms; specific instructions for special needs; connect with caregiver on web; schedule appointment at hospital; provide web-based information	Not reported	Developing a smartphone app which was a tiered tool for self-triage of COVID-19 symptoms was purported to be able to reduce burden on hospitals, provide further self-isolation instructions for users, and to help patients make hospital appointments on web or for virtual visits with health providers, such as with psychologists. The app showed to be a comprehensive tool that may help to reduce spread and panic. The authors suggest further implementation into the popular WeChat app would improve usability.

^a^WHO: World Health Organization.

^b^RT-PCR: reverse transcription polymerase chain reaction.

^c^ED: emergency department.

^d^ICU: intensive care unit.

^e^ER: emergency room.

^f^GP: general practitioner.

^g^IVACS: Intelligent Voice Assistant for Coronavirus Disease (COVID-19) Self-Assessment.

^h^EMS: emergency medical service.

^i^ICT: information and communications technology.

^j^AI: artificial intelligence.

### Integration Into a Health Care System

Of the 22 included studies, 17 (77%) studied self-assessment tools that were integrated within 21 distinct health care systems (Table 1). Many health care systems related to national bodies such as the Iranian Ministry of Health [27], the French national health care system [13,15,26], the Swiss Federal Office of Public Health [14], the Finnish government [33], the Nigerian Center for Disease Control [30], and the United States Centers for Disease Control and Prevention [12]. Kouroubali et al [32] developed a tool to be used throughout Greece. Several health care systems were part of the local health systems involving local institutions such as Mass General Brigham Hospital [19], University of California San Francisco Health [3], and a Canadian university clinic for students [6]. One study compared the quality of triage advice provided among several government-provided tools, including the United States Centers for Disease Control and Prevention Symptom Checker, the United Kingdom 111 COVID-19 Symptom Checker, the Singapore COVID-19 Symptom Checker, and the Japan Stop COVID-19 Symptom Checker [12].

### Administration of Tools

Of the included studies (n=22), a large majority (n=18, 82%) were accessed via the web or an app (including web-based and mobile-based apps). Two studies used a patient portal platform integrated directly into their health care electronic medical record system [11,25], and 1 study created its own platform [36]. One of these tools was accessed via an Amazon Echo [34], and 3 studies developed an artificial intelligence–driven symptom checker [11,19,25], where the participants would put in information and the computer would learn responses to better direct care over time. However, the artificial intelligence technology and use of the tool were still through a website. Three studies described the development of tools that were not yet deployed [29,32,34]. Dhakal et al [34], Yu et al [29], and Heo et al [36] reported the development of a self-triage tool that appears to be independent of any health care system, although the tool in South Korea was reported to be used within the Korean military.

### Direction of Care

Details on the direction of care provided by each tool are presented in Table 2. Commonly, users were advised to go to an emergency room, seek urgent care, contact or see a physician, or stay at home and self-isolate. Several studies described how the direction of care would change depending on the risk of exposure and having disease. For example, in the study by Judson et al [3], asymptomatic patients were asked about their exposure history, and symptomatic patients were triaged to emergent, urgent, nonurgent, or self-care when appropriate or directly connected with a health care provider [3]. One tool offered 8 levels of triage advice [25]. Some directed users to be tested [14,27,36], advised about over-the-counter medications [34], or provided a way to directly contact a caregiver or hotline [3,10,13-15,24,26,31].

### Research Questions and Purposes

The studies included in this scoping review addressed a wide variety of research questions, as presented in Table 2. Overall, 8 studies reported on design, implementation, and use within a broader system [14,19,20,28,30,32,34,36]; 3 studies reported only on design and user testing of tools [29,31,34]; and 9 studies reported on the use of a tool already deployed [11,12,15,24-27,33,35]. Some studies suggested that they would assess whether a self-triage tool reduced demand on care centers or call centers, although not all reported related findings [6,13,27,28]. Some studies set out to assess whether data input by users predicts future demand for care or allows a locality to monitor outbreaks [13,15,24,26,30].

### Studies’ Key Findings

#### Overview

Table 2 presents the key findings for each study. The findings can be organized by the goals suggested in the *Introduction* section, which are to make triage tools accessible on a mass scale; to ensure consistent quality of triage; to provide easily used tools; to enable our ability to survey, monitor, and predict health outcomes from the collected data; and to reduce demand in emergency departments, call centers, and physician offices.

#### The Tools Facilitate Self-triage on a Mass Scale

Tools deployed within countries by public health agencies were used by the public, seemingly on a mass scale. For example, in France, during the first 8 months of the pandemic, 13,000,343 questionnaires were completed, although they could not report the number of users, given a system that encouraged daily use of the tool [15]; >17,000 people used the system in Switzerland in the first 40 days it was available [14]; and a tool developed in Nigeria was used over 4,000,000 times, with 70% of those in Nigeria [30]. The tool developed in Korean was accessed 105,508 times during a 4-week period by people in 141 countries [36]. This key finding suggests that there is a demand for this type of triage during a pandemic. Some of the studies collaborated across countries, which seems to be a good strategy to make the triage accessible on a mass scale [34,35]. In addition, many of the studies incorporated their tool into an already developed health care system, again making the deployment of the tool easier and more likely to reach the larger population (Table 2).

#### Consistency and Quality of Self-triage Is Uncertain

Only one study by Mansab et al [12] examined the quality of the screening and found that each of the 4 tools examined triaged incorrectly at times, for example, not advising to visit the emergency room for sepsis symptoms. Furthermore, 2 of the tools advised seeking clinical care for approximately 80% of the simulations, whereas 2 other tools advised this only half as often. This would mean that some people would be encouraged to go to a health care professional without needing to or that they are advised to self-isolate when they ought to see a caregiver; the outcome depends on what is the medically correct advice. This suggests that at the time of the study, various decision makers and tool designers did not agree on the advice for a given patient and set of symptoms or possible exposure data. Without further study, we cannot know how widespread or important the differences and errors were. Future studies are needed to determine the specificity and sensitivity of the tools, recognizing that it is difficult for a new virus where little information is available.

#### Usability

Only 2 studies directly assessed the usability of a tool [29,34], however, 2 additional studies did report on the demographics using the digital tools, which may indirectly provide information about the usability of the tools [11,33]. Dhakal et al [34] found that the average time to conduct an assessment using the tool was 2 minutes, but nonnative English-speaking individuals had more difficulty understanding it. Kellerman et al [10] designed their tool with input from laypeople of varying age, race, and socioeconomic status to help make the tool more understandable and usable. At least 2 tools were available in multiple languages [30,36], making them more broadly usable.

#### Demand on Health Care System

Yu et al [29] suggested that the purpose of a self-triage tool is to reduce the burden on the hospitals, similar to what other studies reported in this scoping review (Table 2) [6,13,27,28]. However, the studies by Azadnajafabad [27] and Yu et al [29] did not determine whether it actually reduced burden on a health care system. Jensen et al [28] evaluated the demand on the health care system by monitoring the number of calls to a help phone and found no difference when the tool was implemented. Galmiche et al [13] could not determine whether the demand on call centers was affected but argue the fact that hospitalizations increased 10-fold, whereas calls to emergency call centers increased only 23%, suggests the tool did decrease demand on call centers. More work needs to be done to evaluate the tools to determine if they truly reduce the demand on the health care system.

#### Ability to Survey, Monitor, and Predict Health Outcomes From the Collected Data

The studies in this review suggest that digital self-assessment tools can be used to monitor public health and predict demand for care. Jaeger et al [6] concluded that these tools could be used to monitor outbreaks. Runkel et al [24] tracked the number of users on their self-assessment screen and determined the demographics of individuals reporting mild or severe COVID-19 symptoms. The studies out of the France health care system used the self-assessment tool to develop the connection between COVID-19 and the symptoms of anosmia and to predict health care demand [13,15,26].

## Discussion

### Principal Findings

This scoping review identified the literature on self-triage tools that direct or advised care for adults during a pandemic and considered the key outcomes of these studies. We identified 22 studies that explored pandemic self-triage tools, where tools directed the user to appropriate care given their circumstances. It is through this knowledge that we may better understand self-triage tools’ accessibility, quality of guidance, usability, and impacts on public health and health care systems.

### Self-assessment Tools Proliferated During the COVID-19 Pandemic

During the COVID-19 pandemic, there was rapid, global representation of these self-assessment tools, with tools studied in 18 countries, including the United States, France, Iran, Denmark, China, Nigeria, Ireland, Greece, Finland, Canada, Switzerland, South Korea, India, Nepal, Bangladesh, Japan, the United Kingdom, and Singapore. It was reassuring to see that scholarly practices related to COVID-19 self-assessment tools were represented globally, as the pandemic itself was global. This study did not restrict the inclusion of the studies to only COVID-19, but rather any pandemic; however, only 2 of the included studies were from before the COVID-19 pandemic. Access to digital tools for health care management evolved greatly since 2010 and 2011 when SARS and H1N1 were a concern, explaining why there were fewer studies published discussing the use of digital tools during those pandemics, and a greater reliance on digital tools during the COVID-19 pandemic. In addition, the COVID-19 pandemic affected a very large proportion of the global population, much more so than SARS or H1N1 did, further emphasizing the need for a digital self-assessment approach as many health care systems were at times overwhelmed and would not be able to otherwise triage all who sought care in a timely manner.

### Usability, Quality, and Efficacy of the Self-assessment Tools: Much Left Unknown

Few of the tools in this review assessed the usability of the tool, and only 1 tool assessed the quality of triage [12], with results suggesting that digital triage quality during an evolving pandemic is a concern. This is not surprising, given that most tools included were developed when little was known about COVID-19; this does suggest a need for assessing the quality of self-assessment tools for conditions that are well understood. None of the studies assessed the quality of information provided, for example, the quality of information on how to self-isolate. Many of the tools we reported were developed quickly at the start of the pandemic and deployed for use. The lack of research on the quality of triage and advice provided, or perhaps comparing digital triage to human triage, means that we do not know whether the end users received optimal triage and information or the same advice they would have received from a caregiver, making it unclear whether the availability of self-triage translated to equivalent or better management of the virus.

The purpose of the self-assessment tools was to monitor and triage symptoms or exposure, disseminate information, reduce the strain on the health care system, and help patients navigate the health care system. Although the purpose of the tools was clearly articulated, few studies tried to empirically assess whether the tools do in fact help with monitoring outbreaks, reduce the strain on the health care system, or help patients navigate the health care system, which are the stated goals of such tools. Many studies reported on the number of users of the application or number of times it was used, but it is not clear how well use translated to managing the pandemic. Future public health studies should assess whether a digital-first approach to triage an impact on reducing viral spread or demands on health care systems. However, it is challenging to empirically measure these outcomes, as there is no way to conduct controlled studies, especially while monitoring the results from a pandemic. A digital-first approach to health care could be a feasible option for the future of health care, but the efficacy and effect of the tools needs to be further investigated.

### Public Health Implications

This scoping review suggests that a digital-first self-assessment may improve the efficiency of the health care system and potentially allow for a greater number of patients to be seen by their health care provider. Junior family physicians often express their concern that they are overworked, underpaid, and undervalued [37]. Family physicians carry a substantial portion of patient care by providing primary care, obstetric, emergency, hospital, palliative, geriatric, and other health services [24,30,38]. Most rural communities rely solely on family physicians [38,39]. It may be possible to reduce the burden on family physicians by introducing a digital-first tool that could help to self-assess or triage patients. It may also offer opportunities to rural communities where access to a family physician could be limited. Further work would have to be done to allow for alternative options for digital self-assessment (it cannot completely eradicate in-person visits as this would marginalize those who do not have access to digital tools) and consider the legal and health care risks that could come with misclassifying an individual [32]. Other limitations of the successful integration of self-triage tools into health care systems might include literacy, language, beliefs, economics, and technology proficiency, which would have to be addressed before a digital self-assessment tool could be fully and equitably integrated into a health care system.

### Integration of the Tools Into the Health Care System

A potential strategy to transition to a more digital approach to health care is to integrate the digital tools into already defined health care systems. In the current scoping review, 17 of the included studies integrated their tool into a defined health care system, meaning that the tool was used in an already established system such as the Ministry of Health, medical services, or public health office. Some of the tools were integrated at the national level, whereas others were integrated at the provincial or local level or within a specific hospital or clinic. It seemed that having the tool integrated into the health care system helped to disseminate the tool to the users and provided some trust to the user [29]. It seems that using the infrastructure of an already established health care program is likely the most effective way to implement a new tool intended to direct care on a large scale, which is likely why most of the studies identified in this scoping examined tools that were integrated into a health care system.

### Strengths and Limitations

This study had several strengths and limitations. This scoping review acknowledges the breadth of literature on digital self-assessment tools through July 15, 2021. A rigorous methodological approach was used and the results were compiled systematically. This study is limited by the fact that some interpretation is required when compiling and summarizing the results. Finally, although a research librarian (MS) conducted the literature search, it is possible that some relevant articles may have been missed in this step. Furthermore, although many steps were taken to avoid this, it is possible that some articles were inappropriately screened out.

### Conclusions

In conclusion, this scoping review identified the literature on a digital approach to health care during a pandemic, specifically examining the literature on self-triage. There is clear interest in pandemic self-triage, given the global development and deployment of self-triage tools during the COVID-19 pandemic and based on use when made available. These tools have been implemented in time of worldwide pandemic crisis, with the nature of the disease changing regularly. There is some evidence that such tools can be used to collect data for monitoring and possibly predicting needs; they can be integrated into existing systems, making triage more accessible. We found no clear evidence that the tools affect demand on the health care system; assessing this question is challenged by the inability to perform controlled studies. This is a nascent research domain with many unanswered questions. Given that this scoping review was limited to research published in the first year and a half of the COVID-19 pandemic, these findings must be considered preliminary and suggestive of further research needs. Importantly, there continues to be a need for assessing quality of triage provided by these tools.

## Appendix

Multimedia Appendix 1 Search strategies.

## Abbreviations

PRISMA: Preferred Reporting Items for Systematic Reviews and Meta-Analyses
SARS: severe acute respiratory syndrome

## Data Availability

Data from this study are available on the web from the original manuscripts that were included in this review.

## References

[1] Keesara S, Jonas A, Schulman K (2020). COVID-19 and health care's digital revolution. N Engl J Med.

[2] Hollander JE, Carr BG (2020). Virtually perfect? Telemedicine for COVID-19. N Engl J Med.

[3] Judson TJ, Odisho AY, Neinstein AB, Chao J, Williams A, Miller C, Moriarty T, Gleason N, Intinarelli G, Gonzales R (2020). Rapid design and implementation of an integrated patient self-triage and self-scheduling tool for COVID-19. J Am Med Inform Assoc.

[4] Anhang Price R, Fagbuyi D, Harris R, Hanfling D, Place F, Taylor TB, Kellermann AL (2013). Feasibility of web-based self-triage by parents of children with influenza-like illness: a cautionary tale. JAMA Pediatr.

[5] Drew DA, Nguyen LH, Steves CJ, Menni C, Freydin M, Varsavsky T, Sudre CH, Cardoso MJ, Ourselin S, Wolf J, Spector TD, Chan AT, COPE Consortium (2020). Rapid implementation of mobile technology for real-time epidemiology of COVID-19. Science.

[6] Jaeger V, Shick-Porter M, Moore D, Grant D, Wolfe V (2011). GotFlu channel: an online syndromic surveillance tool supporting college health practice and public health work. J Am Coll Health.

[7] Hswen Y, Brownstein JS, Xu X, Yom-Tov E (2020). Early detection of COVID-19 in China and the USA: summary of the implementation of a digital decision-support and disease surveillance tool. BMJ Open.

[8] Lazer D, Kennedy R, King G, Vespignani A (2023). Google Flu Trends still appears sick: an evaluation of the 2013-2014 flu season. SSRN J. Preprint posted online on March 13, 2014.

[9] Wagner M, Lampos V, Yom-Tov E, Pebody R, Cox IJ (2017). Estimating the population impact of a new pediatric influenza vaccination program in England using social media content. J Med Internet Res.

[10] Kellermann AL, Isakov AP, Parker R, Handrigan MT, Foldy S (2010). Web-based self-triage of influenza-like illness during the 2009 H1N1 influenza pandemic. Ann Emerg Med.

[11] Perlman A, Vodonos Zilberg A, Bak P, Dreyfuss M, Leventer-Roberts M, Vurembrand Y, Jeffries HE, Fisher E, Steuerman Y, Namir Y, Goldschmidt Y, Souroujon D (2020). Characteristics and symptoms of app users seeking COVID-19-related digital health information and remote services: retrospective cohort study. J Med Internet Res.

[12] Mansab F, Bhatti S, Goyal D (2021). Performance of national COVID-19 'symptom checkers': a comparative case simulation study. BMJ Health Care Inform.

[13] Galmiche S, Rahbe E, Fontanet A, Dinh A, Bénézit F, Lescure FX, Denis F (2020). Implementation of a self-triage web application for suspected COVID-19 and its impact on emergency call centers: observational study. J Med Internet Res.

[14] Hautz WE, Exadaktylos A, Sauter TC (2021). Online forward triage during the COVID-19 outbreak. Emerg Med J.

[15] Denis F, Fontanet A, Le Douarin YM, Le Goff F, Jeanneau S, Lescure FX (2021). A self-assessment web-based app to assess trends of the COVID-19 pandemic in France: observational study. J Med Internet Res.

[16] Eldh AC, Sverker A, Bendtsen P, Nilsson E (2020). Health care professionals' experience of a digital tool for patient exchange, anamnesis, and triage in primary care: qualitative study. JMIR Hum Factors.

[17] Rodgers M, Raine G, Thomas S, Harden M, Eastwood A (2019). Informing NHS policy in 'digital-first primary care': a rapid evidence synthesis. Health Serv Deliv Res.

[18] Definition of Triage. Merriam-Webster.

[19] Lai L, Wittbold KA, Dadabhoy FZ, Sato R, Landman AB, Schwamm LH, He S, Patel R, Wei N, Zuccotti G, Lennes IT, Medina D, Sequist TD, Bomba G, Keschner YG, Zhang HM (2020). Digital triage: novel strategies for population health management in response to the COVID-19 pandemic. Healthc (Amst).

[20] Arksey H, O'Malley L (2005). Scoping studies: towards a methodological framework. Int J Soc Res Methodol.

[21] Colquhoun HL, Levac D, O'Brien KK, Straus S, Tricco AC, Perrier L, Kastner M, Moher D (2014). Scoping reviews: time for clarity in definition, methods, and reporting. J Clin Epidemiol.

[22] Levac D, Colquhoun H, O'Brien KK (2010). Scoping studies: advancing the methodology. Implement Sci.

[23] Page MJ, McKenzie JE, Bossuyt PM, Boutron I, Hoffmann TC, Mulrow CD, Shamseer L, Tetzlaff JM, Akl EA, Brennan SE, Chou R, Glanville J, Grimshaw JM, Hróbjartsson A, Lalu MM, Li T, Loder EW, Mayo-Wilson E, McDonald S, McGuinness LA, Stewart LA, Thomas J, Tricco AC, Welch VA, Whiting P, Moher D (2021). The PRISMA 2020 statement: an updated guideline for reporting systematic reviews. BMJ.

[24] Runkle JD, Sugg MM, Graham G, Hodge B, March T, Mullendore J, Tove F, Salyers M, Valeika S, Vaughan E (2021). Participatory COVID-19 surveillance tool in rural Appalachia: real-time disease monitoring and regional response. Public Health Rep.

[25] Morse KE, Ostberg NP, Jones VG, Chan AS (2020). Use characteristics and triage acuity of a digital symptom checker in a large integrated health system: population-based descriptive study. J Med Internet Res.

[26] Denis F, Galmiche S, Dinh A, Fontanet A, Scherpereel A, Benezit F, Lescure FX (2020). Epidemiological observations on the association between anosmia and COVID-19 infection: analysis of data from a self-assessment web application. J Med Internet Res.

[27] Azadnajafabad S, Saeedi Moghaddam S, Rezaei N, Ghasemi E, Naderimagham S, Azmin M, Mohammadi E, Jamshidi K, Fattahi N, Zokaei H, Mehregan A, Damerchilu B, Fathi P, Erfani H, Norouzinejad A, Gouya MM, Jamshidi H, Malekzadeh R, Larijani B, Farzadfar F (2021). A report on statistics of an online self-screening platform for COVID-19 and its effectiveness in Iran. Int J Health Policy Manag.

[28] Jensen T, Holgersen MG, Jespersen MS, Blomberg SN, Folke F, Lippert F, Christensen HC (2021). Strategies to handle increased demand in the COVID-19 crisis: a coronavirus EMS support track and a web-based self-triage system. Prehosp Emerg Care.

[29] Yu J, Zhang HW, Shao Y, Lei Y, Chen H, Pu ZH, Lin F, Xu HJ, Wang YL, Liang C, Liu LH, Liu XJ, Dai WC (2020). A smartphone-based online tool for prehospital self-triage of COVID-19. Chin J Acad Radiol.

[30] Owoyemi A, Ikpe R, Toye M, Rewane A, Abdullateef M, Obaseki E, Mustafa S, Adeosun W (2021). Mobile health approaches to disease surveillance in Africa; Wellvis COVID triage tool. Digit Health.

[31] Lunn PD, Timmons S, Julienne H, Belton CA, Barjaková M, Lavin C, McGowan FP (2021). Using decision aids to support self-isolation during the COVID-19 pandemic. Psychol Health.

[32] Kouroubali A, Kondylakis H, Kavlentakis G, Logothetides F, Stathiakis N, Petrakis Y, Tzikoulis V, Kostomanolakis S, Katehakis DG (2020). An eHealth platform for the holistic management of COVID-19. Stud Health Technol Inform.

[33] Jormanainen V, Soininen L (2021). Use and users of the web-based Omaolo COVID-19 symptom self-assesment tool in Finland since March 16, 2020. Stud Health Technol Inform.

[34] Dhakal P, Damacharla P, Javaid AY, Vege HK, Devabhaktuni VK (2020). IVACS: I ntelligent V oice A ssistant for C oronavirus disease (COVID-19) S elf-assessment. Proceedings of the 2020 International Conference on Artificial Intelligence & Modern Assistive Technology.

[35] Collado-Borrell R, Escudero-Vilaplana V, Villanueva-Bueno C, Herranz-Alonso A, Sanjurjo-Saez M (2020). Features and functionalities of smartphone apps related to COVID-19: systematic search in app stores and content analysis. J Med Internet Res.

[36] Heo J, Sung M, Yoon S, Jang J, Lee W, Han D, Kim HJ, Kim HK, Han JH, Seog W, Ha B, Park YR (2020). A patient self-checkup app for COVID-19: development and usage pattern analysis. J Med Internet Res.

[37] Limb M (2015). New contract would mean doctors are "overworked, underpaid, undervalued, demoralised". BMJ.

[38] Ginzburg VE (2007). Feeding stereotypes. Can Fam Physician.

[39] Gorsky K, Safran T (2017). Small town, big picture: scope of practice of rural family medicine, the Shawville experience. Mcgill J Med.

