# Supplementary material for: A Digital-First Health Care Approach to Managing Pandemics: Scoping Review of Pandemic Self-triage Tools
Source: J Med Internet Res. 2023 May 17;25:e40983. doi: 10.2196/40983 (PMC10198718; doi:10.2196/40983)
Supplement: Multimedia Appendix 1 [file jmir_v25i1e40983_app1.docx]

APPENDIX A

1. Selection criteria

[Insert inclusion and exclusion criteria that the screeners used]

[Insert finalized PICO chart]

Studies discussing an element of self-triage, related to COVID-19 and other global pandemics were selected, following the Arskey and O’Mally framework.

P- adults

I- pandemic triage tools that guide towards direct care

C- doesn’t necessarily need to have a control, can be an observational study. But could be telephone or face to face appointments

O- safety, clinical effectiveness, cost effectiveness, accuracy

1. Search

MEDLINE, Embase, Scopus, PsycINFO, CINAHL and Cochrane were all searched on July 14^th^ and July 15^th^, 2021. These databases were selected due to the nature of their peer reviewed journal content relating to the research question. Because we were seeking research on COVID-19, and therefore seeking research as it was rapidly being produced, we included the consideration of conference proceedings, pre-prints, non-English language articles, and any other formats retrieved in our searches. No limits were applied to the search that would restrict the results to exclude these.

| MEDLINE Search History: |
| --- |
|  |
| 1. triage.tw. or Triage/ |
| 1. self-triage.tw. |
| 1. screening tool*.tw. |
| 1. Self-Assessment/ or self assessment tool*.tw. |
| 1. symptom check*.tw. |
| 1. self evaluation.tw. |
| 1. Decision Support Techniques/ or decision support tool*.tw. |
| 1. 1 or 2 or 3 or 4 or 5 or 6 or 7 |
| 1. telemedicine.tw. or Telemedicine/ |
| 1. internet.tw. or Internet/ or Internet-Based Intervention/ |
| 1. web-based.tw. |
| 1. Online Systems/ or online.tw. |
| 1. mobile health.tw. |
| 1. telehealth.tw. |
| 1. ehealth.tw. |
| 1. telephone.tw. or Telephone/ |
| 1. mobile application.tw. or Mobile Applications/ |
| 1. 9 or 10 or 11 or 12 or 13 or 14 or 15 or 16 or 17 |
| 1. exp COVID-19/ or COVID.tw. |
| 1. coronavirus.tw. or Coronavirus/ |
| 1. SARS Virus/ or SARS-CoV-2/ or SARS.tw. |
| 1. Influenza A Virus, H1N1 Subtype/ or H1N1.tw. |
| 1. pandemic.tw. or Pandemics/ |
| 1. 19 or 20 or 21 or 22 or 23 |
| 1. 8 and 18 and 24 |

| **Keywords:** |
| --- |
| *Self-Triage:* |
| “self triage” OR triage OR “screening tool*” OR “self assessment tool*” OR “symptom check*” OR “self evaluation” OR “decision support tool*” |
|  |
| *Mobile Health:* |
| “mobile health” OR telemedicine OR internet OR “web based” OR online OR telehealth OR ehealth OR telephone OR “mobile application*” |
|  |
| *COVID-19:* |
| “covid” OR coronavirus OR SARS OR H1N1 OR pandemic |
|  |
| **MeSH Terms:** |
| *Self-Triage:* |
| Triage/ OR Self-Assessment/OR Decision Support Techniques/ |
|  |
| *Mobile Health:* |
| Telemedicine/ OR Internet/ or Internet-Based Intervention/ OR Online Systems/ OR Telephone/ OR Mobile Applications/ |
|  |
| *COVID-19:* |
| Exp.COVID-19/ OR Coronavirus/ OR SARS Virus/ or SARS-CoV-2/ OR Influenza A Virus, H1N1 Subtype/ |
|  |

*The

Database Search Results for individual databases:

| Database | Number of Results |
| --- | --- |
| MEDLINE | 554 |
| EMBASE | 742 |
| Scopus | 656 |
| PsycINFO | 41 |
| CINAHL | 407 |
| Cochrane | 16 |
